# Interpretive Reference Ranges for Plasma Drug Concentrations in Acute Recreational Drug Toxicity: A Short Communication

**DOI:** 10.1097/FTD.0000000000001471

**Published:** 2026-04-23

**Authors:** Stefania Boccuzzi, Dilek Battal, David Cowan, Paul I. Dargan, Vincenzo Abbate

**Affiliations:** * Department of Analytical, Environmental and Forensic Sciences, Faculty of Life Sciences and Medicine, King's College London, London, United Kingdom;; † Department of Toxicology, Faculty of Pharmacy, Mersin University, Mersin, Turkey;; ‡ Clinical Toxicology, Faculty of Life Sciences and Medicine, King's College London, London, United Kingdom; and; § Clinical Toxicology, Guy's and St Thomas' NHS Foundation Trust and King's Health Partners, London, United Kingdom.

**Keywords:** acute recreational drug toxicity, clinical toxicology, plasma drug concentrations, interpretive guidelines

## Abstract

**Background:**

Acute recreational drug toxicity (ARDT) is a frequent cause of emergency department presentations; however, clinicians lack guidance for interpreting drug concentrations. Existing terminology (“therapeutic,” “toxic,” and “lethal”) does not reflect the variability observed in ARDT, where tolerance, interindividual differences, and polydrug exposure influence clinical features. This study aimed to develop a descriptive, clinician-oriented framework to categorize illicit and prescription drug concentrations into meaningful interpretive bands.

**Methods:**

A literature search identified studies reporting antemortem plasma concentrations in adults. Twenty-four analytes (parent drugs and metabolites) commonly detected in ARDT were included. Concentrations were standardized to ng/mL. For illicit drugs, skewness, histograms, and robust z-scores (median absolute deviation–based; threshold ±3.5) were used to identify and remove outliers. Interquartile- or tertile-based interpretive bands (low, moderate, and high) were assigned according to data set characteristics. Gamma-hydroxybutyrate concentrations were classified using endogenous cutoff values. Prescription medicines were categorized as within or above the therapeutic range based on established reference ranges.

**Results:**

Thousand two hundred sixteen plasma concentrations from 79 studies met the inclusion criteria. Illicit drug ranges included analytes with narrow (ketamine, 53–86 ng/mL) and broad (cocaine, 65–193 ng/mL) concentration–effect variability. The endogenous cutoff for gamma-hydroxybutyrate was 5000 ng/mL. Prescription medicine ranges reflected expected therapeutic patterns.

**Conclusions:**

This study proposes a reproducible, percentile-based framework for interpreting plasma drug concentrations in ARDT. The framework provides clinically intuitive, data-driven reference bands to support clinical interpretation, enhance communication between laboratories and clinicians, and offer a scalable foundation for future guideline development.

## Abstract 2

Supplemental Digital Content is Available in the Text.

## INTRODUCTION

Approximately 3 million people reported drug use in England and Wales between April 2023 and March 2024.^1^ Although these rates have remained relatively stable since 2014, hospital admissions for drug poisoning continue to represent a consistent clinical burden. In England alone, 9690 hospital admissions related to drug poisoning occurred between April 2022 and March 2023, equating to 17.3 per 100,000 population.^2^ This figure represents inpatient admissions rather than hospital presentations; therefore, the true burden of acute recreational drug toxicity (ARDT) presentations is likely higher, as many are managed and discharged from the emergency department (ED) without requiring inpatient admission.^3^ At present, no interpretive guidelines define whether a measured plasma drug concentration indicates mild, moderate, or severe exposure, reducing the clinical utility of quantitative results. Instead, clinicians must extrapolate from isolated case reports, pharmacokinetic studies, or forensic data sets.^4^ Stratifying plasma drug concentrations using traditional toxicological terminology of “therapeutic,” “toxic,” and “lethal” is poorly suited to ED settings. These descriptors present challenges because drug tolerance^5^ and interindividual variability^6^ strongly influence physiological responses to drugs. Furthermore, polydrug use significantly affects both clinical risk and the features observed in ARDT presentations.^7^ For example, a plasma concentration considered “toxic” in a drug-naïve patient may be tolerated by a chronic user, whereas a “therapeutic” concentration may precipitate significant adverse effects in individuals with metabolic polymorphisms or organ dysfunction. Similarly, the term “lethal” concentration can be misleading, as fatality depends on many factors beyond the measured drug concentration, including combined drug exposure, medical comorbidities, and the time from clinical deterioration to initiation of treatment.^8^ These limitations underscore the need for terminology that better reflects the clinical realities of ARDT presentations.

To address this gap, this study proposes a descriptive clinician-facing framework based on antemortem data from multiple contexts to provide clinically meaningful reference bands for interpreting plasma drug concentrations in acute care. For illicit drugs, plasma concentrations are classified as low, moderate, or high based on percentile thresholds derived from published data. For prescription medicines, concentrations are described as within or above their therapeutic range. These guidelines aim to provide a pragmatic interpretive structure that improves communication between laboratories and clinicians while acknowledging biological variability and clinical context.

## MATERIALS AND METHODS

A comprehensive literature search was conducted to identify literature sources reporting antemortem plasma concentrations of analytes associated with ARDT. Several analytes included in this study may be encountered in both therapeutic and nonmedical contexts; therefore, categorization was based on an interpretive framework rather than legal or prescription status. Substances with defined therapeutic ranges are referred to as “prescription medicines” whereas those without established therapeutic ranges are referred to as “illicit.” In this study, “antemortem” refers to plasma samples obtained from living patients, in contrast to postmortem toxicology data. These definitions are used throughout the article for clarity. Data were assembled through an iterative literature compilation using predefined inclusion and exclusion criteria, explicit reporting of all included analytes, and full citation of all data sources contributing to the final data set. Duplicate data were assessed during the data extraction stage, and redundant records were excluded to avoid miscounting of plasma concentrations. A literature search was conducted using the PubMed, Scopus, and Google Scholar databases, covering publications from January 1980 to October 2025. Search terms included “plasma concentration,” “toxicology,” “antemortem,” “case report,” and “overdose,” in various combinations with individual drug names. The reference lists of the retrieved articles were manually screened to identify additional studies. The inclusion criteria were as follows: (1) human studies or case reports involving adults (18 years and older), (2) quantitative plasma concentrations confirmed using chromatographic techniques (eg, LC-MS/MS or GC-MS), and (3) antemortem data reported across clinical, forensic, and controlled settings, selected to inform the interpretation of quantitative results encountered in ARDT presentations. The exclusion criteria were as follows: (1) postmortem data, (2) nonhuman or in vitro studies, (3) pediatric reports, and (4) reports limited to nonplasma matrices. For each eligible study, the following information was extracted and recorded: drug name, plasma concentration, and any relevant contextual notes (eg, the dose administered in controlled studies). All data were collated, and concentrations were converted to ng/mL to ensure comparability.

Twenty-four analytes comprising parent drugs and clinically informative metabolites were included in this study. Selection was based on routine antemortem plasma testing and input from consultant toxicologists regarding substances of particular relevance in acute care: delta-9-tetrahydrocannabinol (Δ9-THC), 11-nor-9-carboxy-delta-9-tetrahydrocannabinol (11-COOH-THC), 11-hydroxy-delta-9-tetrahydrocannabinol (11-OH-THC), 6-monoacetylmorphine (6-MAM), alprazolam, amphetamine, buprenorphine, benzoylecgonine (BZE), cocaine, codeine, diazepam, 2-ethylidene-1,5-dimethyl-3,3-diphenylpyrrolidine (EDDP), ethyl glucuronide (EtG), gabapentin, gamma-hydroxybutyrate (GHB), ketamine, 3,4-methylenedioxyamphetamine (MDA), 3,4-methylenedioxymethamphetamine (MDMA), methadone, methamphetamine, morphine, pregabalin, tramadol, and zopiclone. Prescription medicines were classified as “within therapeutic range” or “above therapeutic range” based on distributions reported in pharmacokinetic data and controlled administration studies. The upper boundary of the “within therapeutic range” category reflects the highest concentrations observed following therapeutic dosing. Concentrations exceeding this range, commonly reported in cases of illicit use or overdose, were classified as “above therapeutic range.” Illicit substances were categorized as “low,” “moderate,” or “high” based on percentile-derived thresholds. All statistical analyses of drug concentrations were performed using Microsoft Excel (version 16.101.2; Microsoft Corporation, Redmond, WA). The distribution shape was assessed using skewness estimates and visual inspection of histograms constructed with consistent bin widths to evaluate asymmetry and right skew. Outliers were identified using robust z-scores based on the median, the median absolute deviation (MAD), and the scaled MAD (MAD × 1.4826).^9,10^ Robust z-scores exceeding ±3.5 were treated as outliers and removed before percentile calculation. Interquartile thresholds (P25/P75) were applied when one or more of the following criteria were met: (1) sample size (n < 30), (2) skewness >2, or (3) analytes with steep concentration–effect relationships or narrow exposure ranges. Tertile thresholds (P33/P66) were applied to larger data sets (n ≥ 30) with broader and more evenly distributed variability in observed plasma concentrations. Threshold selection was guided by predefined considerations related to concentration range, breadth, and distribution characteristics, consistent with established clinical toxicology interpretive practices. An example is provided in **Supplemental Digital Content 1** (see **Supplementary Table 1**, http://links.lww.com/TDM/A938). To assess the impact of outlier exclusion, sensitivity analyses were performed by recalculating the descriptive statistics before and after outlier removal. A representative example is provided in **Supplemental Digital Content 1** (see **Supplementary Table 2**, http://links.lww.com/TDM/A938).

## RESULTS

A total of 79 studies met the inclusion criteria, contributing 1216 antemortem plasma concentration values across 24 analytes (Table 1). Of these, 47 studies (n = 697) involved illicit substances, whereas 32 studies (n = 519) described prescription medicines. The studies spanned diverse contexts, including ED presentations (n = 10), intensive care admissions (n = 3), drug-impaired driving evaluations (n = 4), controlled administration studies (n = 21), and therapeutic drug monitoring (n = 23). The compiled data covered a broad range of plasma concentrations, from sub-ng/mL to mg/mL, reflecting differences in pharmacokinetic properties, dose, route of administration, analytical sensitivity, and sampling timing across cases.

**TABLE 1.** Summary of Published Literature Used to Compile Plasma Concentration Data

Category	No. of Studies	Plasma Concentrations (n)	Drugs Included
Illicit substances	47	697	Δ9-THC, 11-COOH-THC, 11-OH-THC, 6-MAM, amphetamine, BZE, cocaine, EtG, GHB, ketamine, MDA, MDMA, methamphetamine, morphine
Prescription medicines	32	519	Alprazolam, buprenorphine, codeine, diazepam, EDDP, gabapentin, methadone, pregabalin, tramadol, zopiclone

Δ9-THC, delta-9-tetrahydrocannabinol; 11-COOH-THC, 11-nor-9-carboxy-delta-9-tetrahydrocannabinol; 11-OH-THC, 11-hydroxy-delta-9-tetrahydrocannabinol; 6-MAM, 6-monoacetylmorphine; BZE, benzoylecgonine; EtG, ethyl glucuronide; GHB, gamma-hydroxybutyrate; MDA, 3,4-methylenedioxyamphetamine; MDMA, 3,4-methylenedioxymethamphetamine; EDDP, 2-ethylidene-1,5-dimethyl-3,3-diphenylpyrrolidine.

Outliers identified using robust z-score criteria (exceeding ±3.5) accounted for a small proportion of data points for each analyte. Outlier removal was not required for all analytes and had variable effects depending on the degree of right skew, with larger effects observed in data sets with extended upper tails. Sensitivity analysis showed that including extreme values increased the upper percentile boundaries for some analytes; therefore, robust outlier exclusion was retained to prevent isolated extreme values from disproportionally influencing threshold selection. Table 2 summarizes the derived interpretive ranges based on these data sets. Morphine, which exhibited a wide range of reported concentrations but a narrow therapeutic window, was better represented using interquartile thresholds (P25 = 12 ng/mL; P75 = 184 ng/mL). By contrast, drugs with broader concentration–effect profiles and larger sample sizes, such as cocaine and MDMA, were more appropriately categorized using tertile cutoffs (P33/P66), yielding balanced subdivisions of low, moderate, and high exposure ranges. For illicit substances, thresholds ranged from low nanogram levels for potent analytes such as 6-MAM (low <5 ng/mL) to hundreds of ng/mL for compounds with higher dose tolerance, such as BZE and cocaine (low <124 and <104 ng/mL, respectively). These ranges reflect real-world variability while maintaining clinical interpretability. GHB was treated as a unique case because of its endogenous presence. A plasma cutoff of 5000 ng/mL, derived from established toxicological literature, was applied to distinguish endogenous concentrations from exogenous exposure rather than using percentile-based thresholds.^11–13^ For prescription medicines, the upper thresholds do not represent absolute toxic limits but rather contextual reference bands. For example, diazepam concentrations ≤1500 ng/mL were classified as therapeutic, whereas values >1500 ng/mL indicated supratherapeutic exposure. Similar cutoff values were identified for the remaining prescription medicines included in this study.

**TABLE 2.** Suggested Interpretive Antemortem Plasma Concentration Ranges for 24 Illicit Substances and Prescription Medicines

Substance	Interpretive Range (ng/mL)	Plasma Concentrations (n)	Threshold Method	References
Illicit substances				
Δ9-THC	Low: <3 Moderate: ≥3 to ≤15 High: >15	154	Interquartile (P25/P75)	14–22
11-COOH-THC	Low: <12 Moderate: ≥12 to ≤43 High: >43	58	Interquartile (P25/P75)	14,15,18–20,22
11-OH-THC	Low: <1 Moderate: ≥1 to ≤3 High: >3	42	Interquartile (P25/P75)	14,18–20,22
6-MAM	Low: <5 Moderate: ≥5 to ≤22 High: >22	27	Interquartile (P25/P75)	14,23–29
Amphetamine	Low: <26 Moderate: ≥26 to ≤77 High: >77	18	Interquartile (P25/P75)	14,20,30,31
BZE	Low: <124 Moderate: ≥124 to ≤333 High: >333	54	Tertile (P33/P66)	14,20,29,32–38
Cocaine	Low: <104 Moderate: ≥104 to ≤253 High: >253	68	Tertile (P33/P66)	14,20,29,32–39
EtG	Low: <280 Moderate: ≥280 to ≤800 High: >800	6	Interquartile (P25/P75)	40,41
GHB	Endogenous: ≤5000 Exogenous: >5000	69	Literature-defined cut-off	11–14,42–50
Ketamine	Low: <53 Moderate: ≥53 to ≤86 High: >86	32	Tertile (P33/P66)	14,51–53
MDA	Low: <6 Moderate: ≥6 to ≤20 High: >20	22	Interquartile (P25/P75)	14,20,30,54–56
MDMA	Low: <65 Moderate: ≥65 to ≤193 High: >193	34	Tertile (P33/P66)	14,20,30,54–56
Methamphetamine	Low: <20 Moderate: ≥20 to ≤65 High: >65	16	Interquartile (P25/P75)	14,20,31,57
Morphine	Low: <12 Moderate: ≥12 to ≤184 High: >184	97	Tertile (P33/P66)	14,20,23–29,58–60
Prescription medicines				
Alprazolam	Within therapeutic range: ≤154 Above therapeutic range: >154	34	Therapeutic-range based	14,61
Buprenorphine	Within therapeutic range: ≤175 Above therapeutic range: >175	53	Therapeutic-range based	14,62–66
Codeine	Within therapeutic range: ≤340 Above therapeutic range: >340	66	Therapeutic-range based	14,20,29,34,60,67,68
Diazepam	Within therapeutic range: ≤1500 Above therapeutic range: >1500	30	Therapeutic-range based	14,69,70
EDDP	Within therapeutic range: ≤176 Above therapeutic range: >176	30	Therapeutic-range based	29,71–74
Gabapentin	Within therapeutic range: ≤8900 Above therapeutic range: >8900	12	Therapeutic-range based	14,75,76
Methadone	Within therapeutic range: ≤1530 Above therapeutic range: >1530	45	Therapeutic-range based	14,29,71–73
Pregabalin	Within therapeutic range: ≤9425 Above therapeutic range: >9425	22	Therapeutic-range based	14,77–83
Tramadol	Within therapeutic range: ≤345 Above therapeutic range: >345	21	Therapeutic-range based	14,84
Zopiclone	Within therapeutic range: ≤131 Above therapeutic range: >131	13	Therapeutic-range based	14,85–87

The descriptors “low,” “moderate,” and “high” reflect relative positions within published antemortem plasma concentration distributions, and do not imply clinical severity. Threshold selection accounted for both data set size and concentration distribution. Although larger data sets typically permitted tertile thresholds, interquartile thresholds were retained when concentration ranges were narrow or exhibited steep exposure–effect relationships, as broader partitioning would have reduced interpretability.

Δ9-THC, delta-9-tetrahydrocannabinol; 11-COOH-THC, 11-nor-9-carboxy-delta-9-tetrahydrocannabinol; 11-OH-THC, 11-hydroxy-delta-9-tetrahydrocannabinol; 6-MAM, 6-monoacetylmorphine; BZE, benzoylecgonine; EtG, ethyl glucuronide; GHB, gamma-hydroxybutyrate; MDA, 3,4-methylenedioxyamphetamine; MDMA, 3,4-methylenedioxymethamphetamine; EDDP, 2-ethylidene-1,5-dimethyl-3,3-diphenylpyrrolidine.

## DISCUSSION

This study presents an adaptable, statistically driven framework for interpreting plasma drug concentrations in acute clinical toxicological settings. Using data from 79 published case series and observational studies, percentile-based descriptors were derived to define low, moderate, and high concentration ranges for illicit substances and to classify prescription medicines as within or above the therapeutic range. The resulting interpretive bands represent percentile-based distributions of antemortem plasma concentrations across clinically relevant contexts rather than ARDT-specific toxicity thresholds. This approach reflects the reality that drugs involved in ED presentations often span a continuum from incidental exposure to overt toxicity. Furthermore, the descriptors for illicit substances are intended to convey relative concentration magnitudes rather than clinical severity.

Extreme percentile cutoffs, such as P5/P95, were found to disproportionately reflect rare values and reduce interpretability in acute clinical settings, where clinicians require contextual guidance rather than extreme-case boundaries. The use of percentile-based thresholds mirrors standard practices for establishing clinical reference intervals, in which method selection depends on sample size and data distribution rather than the application of uniform numerical cutoffs.^88^

Plasma concentrations were used because they are routinely measured in hospital laboratories^89^ and form the basis of most pharmacokinetic and toxicological studies.^90^ Postmortem data were excluded because applying such data to antemortem settings can inflate apparent plasma concentrations owing to the absence of an upper bound for postmortem concentrations and the potential for postmortem redistribution.^91,92^ Removal of extreme values was performed to stabilize percentile estimates rather than to exclude clinically severe cases and does not imply the absence of high-risk presentations. Although plasma drug concentrations are influenced by factors such as sampling time, route of administration, formulation, co-exposure, and tolerance, these variables are often unknown at the point of care in ARDT. Therefore, the present framework prioritizes the pragmatic interpretability of measured plasma values over pharmacokinetic modelling, reflecting how quantitative results are used in routine emergency practice.

The proposed categories of “prescription medicines” and “illicit substances” are not intended to define misuse or prescribing suitability, but rather to support rapid, clinically meaningful interpretation of quantitative plasma concentrations alongside patient history and clinical context. Comparison with existing pharmacokinetic and toxicological literature suggests that the proposed interpretive ranges are broadly concordant with previously reported concentration data. For medicinal substances, thresholds classified as “within therapeutic range” aligned with established steady-state or controlled administration values reported in therapeutic drug monitoring and pharmacokinetic studies, whereas concentrations above these thresholds overlapped with those described in overdose or misuse contexts. For illicit substances, percentile-derived “low” concentrations frequently overlapped with values reported in controlled administration or nontoxic exposure studies, whereas “high” ranges encompassed concentrations reported in severe intoxication or overdose case series.^4,14^ These findings support the validity of the proposed descriptors, although formal outcome-based validation remains an important area for future research.

These guidelines are intended as assistive interpretive tools to complement clinical judgment and the pattern of clinical features observed in individual patient presentations. For instance, a low concentration of cocaine in combination with a low concentration of amphetamine in a patient with sympathomimetic features may indicate synergistic cardiovascular risk, enabling clinicians to contextualize results according to relative concern rather than relying on rigid plasma concentration thresholds. The inclusion of pharmacologically inactive metabolites, such as BZE or EtG, as validated biomarkers may also aid clinical interpretation in cases in which the parent compound is no longer detectable. The formal integration of quantitative concentration thresholds with outcome-based models of polydrug toxicity remains an important direction for future research.

Further work could incorporate these ranges into laboratory software, allowing automated interpretive comments to accompany quantitative plasma results and facilitating clearer communication between laboratory staff and clinicians. This framework does not impose a fixed minimum sample size across all analytes. Instead, robustness is addressed through adaptive threshold selection, with interquartile cutoffs applied to smaller or skewed data sets to preserve stability. The analyte panel is not exhaustive but is representative of substances commonly encountered in practice. As such, the statistical approach supports reproducibility and scalability, enabling additional or emerging substances to be incorporated using the same decision logic.

Interpretation bands are based on antemortem plasma concentrations derived from published data, which vary in sampling time, analytical detection methods, and patient characteristics—factors known to introduce variability in measured drug levels.^93^ Although the percentile-based approach mitigates some of this heterogeneity, it cannot limit it entirely. A key limitation is that the included plasma concentrations were not restricted to symptomatic ARDT cases and therefore did not permit stratification by clinical severity at the time of sampling. The proposed ranges should therefore be interpreted as contextual reference bands to support clinical judgment rather than as diagnostic thresholds of absolute toxicity.

Future work should focus on expanding this framework to include additional substances and integrating it into clinical settings. Embedding such guidelines into electronic hospital systems could enable automated interpretive reporting,^94^ and collaboration with professional toxicology societies may help establish consensus-driven guidelines. In addition, integrating plasma concentration data with systematically collected clinical outcomes in future research could help determine whether specific concentration bands are associated with higher clinical risk, thereby enabling further refinement of the guidelines.

## CONCLUSIONS

This study proposes a percentile-based interpretive framework for plasma drug concentrations that is statistically transparent, clinically intuitive, and adaptable across diverse analytes. By replacing subjective interpretations and inapplicable “toxic” or “lethal” labels with percentile-derived descriptors, this approach provides a reproducible and transparent method for contextualizing quantitative results in ARDT presentations. The resulting interpretive bands—low, moderate, high/within therapeutic range, and above the therapeutic range—enable clinicians and laboratory professionals to rapidly contextualize plasma results while acknowledging inherent interindividual variability. The framework is not intended as a definitive diagnostic tool but rather as a quantitative guideline that supports clinical judgment and enhances communication between laboratories and clinicians. Future refinement through expanded data sets and integration with electronic health systems could transform this model into a standardized, scalable tool for real-time clinical decision support. Ultimately, these guidelines provide a foundation for developing evidence-based, clinician-facing toxicological interpretation standards applicable across ED settings.

## Supplementary Material
