# Supplementary material for: Interpretive Reference Ranges for Plasma Drug Concentrations in Acute Recreational Drug Toxicity: A Short Communication
Source: Ther Drug Monit. 2026 Apr 23;48(3):409–16. doi: 10.1097/FTD.0000000000001471 (PMC13152082; doi:10.1097/FTD.0000000000001471)
Supplement: Supplementary file 1 [file tdm-48-409-s001.docx]

| **Table S1. Worked example of 11-OH-THC statistics and outlier detection using robust Z scores** | | | | | | | | | |
| --- | --- | --- | --- | --- | --- | --- | --- | --- | --- |
| *Ref.* | *Level (ng/ml)* | *Skewness* | *Histogram distribution* | *Absolute deviation* | *Robust Z* | *Outlier?* | *Median* | *MAD* | *Scaled MAD* |
| ^21^ | 6.1 | 1.88 | Right skewed | 4.24 | 1.97 | No | 1.87 | 1.45 | 2.15 |
|  | 2.8 |  |  | 0.94 | 0.43 | No |  |  |  |
|  | 20.0 |  |  | 18.14 | 8.44 | Yes |  |  |  |
|  | 20.0 |  |  | 18.14 | 8.44 | Yes |  |  |  |
|  | 4.1 |  |  | 2.24 | 1.04 | No |  |  |  |
|  | 7.0 |  |  | 5.14 | 2.39 | No |  |  |  |
|  | 3.3 |  |  | 1.44 | 0.67 | No |  |  |  |
|  | 10.0 |  |  | 8.14 | 3.78 | Yes |  |  |  |
|  | 0.7 |  |  | 1.17 | -0.54 | No |  |  |  |
|  | 3.4 |  |  | 1.54 | 0.71 | No |  |  |  |
|  | 6.9 |  |  | 5.04 | 2.34 | No |  |  |  |
|  | 3.0 |  |  | 1.14 | 0.53 | No |  |  |  |
| ^25^ | 2.6 |  |  | 0.74 | 0.34 | No |  |  |  |
|  | 1.2 |  |  | 0.67 | -0.31 | No |  |  |  |
|  | 0.8 |  |  | 1.07 | -0.50 | No |  |  |  |
|  | 0.7 |  |  | 1.17 | -0.54 | No |  |  |  |
|  | 0.5 |  |  | 1.37 | -0.63 | No |  |  |  |
|  | 0.3 |  |  | 1.57 | -0.73 | No |  |  |  |
|  | 0.2 |  |  | 1.67 | -0.77 | No |  |  |  |
|  | 0.2 |  |  | 1.67 | -0.77 | No |  |  |  |
|  | 0.2 |  |  | 1.67 | -0.77 | No |  |  |  |
|  | 0.2 |  |  | 1.67 | -0.77 | No |  |  |  |
|  | 8.3 |  |  | 6.44 | 2.99 | No |  |  |  |
|  | 4.2 |  |  | 2.34 | 1.09 | No |  |  |  |
|  | 2.4 |  |  | 0.54 | 0.25 | No |  |  |  |
|  | 1.5 |  |  | 0.37 | -0.17 | No |  |  |  |
|  | 1.9 |  |  | 0.03 | 0.02 | No |  |  |  |
|  | 0.8 |  |  | 1.07 | -0.50 | No |  |  |  |
|  | 0.7 |  |  | 1.17 | -0.54 | No |  |  |  |
|  | 0.5 |  |  | 1.37 | -0.63 | No |  |  |  |
|  | 0.4 |  |  | 1.47 | -0.68 | No |  |  |  |
| ^26^ | 1.1 |  |  | 0.75 | -0.35 | No |  |  |  |
|  | 11.1 |  |  | 9.28 | 4.31 | Yes |  |  |  |
|  | 1.8 |  |  | 0.03 | -0.02 | No |  |  |  |
|  | 12.3 |  |  | 10.48 | 4.87 | Yes |  |  |  |
| ^27^ | 0.5 |  |  | 1.35 | -0.63 | No |  |  |  |
|  | 14.2 |  |  | 12.34 | 5.74 | Yes |  |  |  |
| ^29^ | 7.0 |  |  | 5.14 | 2.39 | No |  |  |  |
|  | 0.3 |  |  | 1.57 | -0.73 | No |  |  |  |
|  | 2.0 |  |  | 0.14 | 0.06 | No |  |  |  |
|  | 1.7 |  |  | 0.17 | -0.08 | No |  |  |  |
|  | 1.2 |  |  | 0.67 | -0.31 | No |  |  |  |
| Ref, references; 11-OH-THC, 11-hydroxy-delta-9-tetrahydrocannabinol; MAD, median absolute deviation.  Scaled MAD = MAD $\times$ 1.4826; robust Z = (concentration - median)/scaled MAD. Outliers were defined by robust Z > 3.5 and removed from subsequent percentile assignments. References correspond to those in the main manuscript. | | | | | | | | | |

| **Table S2. Sensitivity analysis for 11-OH-THC: descriptive statistics before and after outlier removal** | | |
| --- | --- | --- |
| *Statistic* | *Value* | |
|  | *Before outlier removal* | *After outlier removal* |
| Total concentration values (n) | 42 | 36 |
| Median (ng/mL) | 1.87 | 1.35 |
| Distribution shape | Right-skewed | Right-skewed |
| Range (min-max, ng/mL) | 0.2 – 20 | 0.2 – 8.3 |
| Percentile thresholds | Interquartile, P25/P75 | Interquartile, P25/P75 |
| Impact on boundary values | Low: < 1  Moderate: ≥ 1 to ≤ 6  High: > 6 | Low: < 1  Moderate: ≥ 1 to ≤ 3  High: > 3 |
